# Essential Oil Inhalation on Blood Pressure and Salivary Cortisol Levels in Prehypertensive and Hypertensive Subjects

**DOI:** 10.1155/2012/984203

**Published:** 2012-11-19

**Authors:** In-Hee Kim, Chan Kim, Kayeon Seong, Myung-Haeng Hur, Heon Man Lim, Myeong Soo Lee

**Affiliations:** ^1^College of Nursing, Eulji University, Daejeon, Republic of Korea; ^2^School of Medicine, Eulji University, Daejeon, Republic of Korea; ^3^Appenzeller School of Public Administration, Paichai University, Daejeon, Republic of Korea; ^4^Medical Research Division, Korea Institute of Oriental Medicine, Daejeon, Republic of Korea

## Abstract

The purpose of this study was to identify the effects of essential oil inhalation on the 24-hour ambulatory blood pressure (BP) and salivary cortisol level in 83 prehypertensive and hypertensive subjects. The experimental group (*n* = 28) was asked to inhale an essential oil blended with lavender, ylang-ylang, marjoram, and neroli (20 : 15 : 10 : 2), whereas the placebo group (*n* = 27) was asked to inhale an artificial fragrance for 24 hours and the control group received no treatment (*n* = 28). The SBP (*P* < .001) and DBP (*P* = .009) measured at home in the experimental group were significantly decreased compared with the placebo group and the control group after treatment. The daytime SBP during the 24-hour ambulatory BP measurement of the experimental group presented with significant decreases in comparison with the measurements of the placebo group and the control group (*P* < .001). There was no statistically significant difference in the nighttime SBPs. The daytime DBPs during the 24-hour ambulatory BP measurements of the experimental group presented with significant decreases in comparison with the measurements of the placebo group and the control group (*P* = .002). There was no significant difference in the night time DBPs. The experimental group showed significant decreases in the concentration of salivary cortisol in comparison with the concentrations of the placebo group and the control group (*P* = .012). In conclusion, the inhalation of an essential oil had immediate and continuous effects on the home SBP, daytime BP, and the stress reduction. Essential oils may have relaxation effects for controlling hypertension.

## 1. Introduction

The prevalence of hypertension in adults older than 30 increased from 2.3% to 24.6% in 2007 to 26.9% in 2008, with males having a higher prevalence than females (29.4% versus 26.4% of females), and the prevalence increases with age. The prevalence of prehypertension is 23.4% overall and is also higher in males (28.4% versus 18.7% of females), with a significant increase in prevalence starting in the 40s [1]. 

The management of hypertension is primarily focused on the use of antihypertensive medications and lifestyle modifications [2], but many patients have difficulty accepting pharmacotherapy because the duration of therapy will be lifelong and because long-term pharmacotherapy will lead to target organ damage. Although the rate of pharmacotherapy is rising, from 18.2% in 1998 to 47.1% in 2005, 58% in 2007, and 59.4% in 2008, this rate is still much lower compared to the 68% in the United States [1]. 

There are many complementary and alternative therapies aimed at lowering the activity of the sympathetic nervous system in patients with hypertension [3]. Aromatherapy has been claimed to be effective in decreasing one's BP, and heart rate using the essential oil of plants [4–8]. However, previous studies measured BP once or twice weekly to monitor physiological changes to assess the antihypertensive effect of essential oils [4–6]. In clinical practice, the diagnosis of hypertension using one or two BP measurements and the subsequent evaluation of a therapeutic effect on the patient leave much room for error. Thus, a 24-hour ambulatory BP monitoring and self-measurement have been advocated [9], and a 24-hour ambulatory BP monitoring is recommended for evaluating the BP control of patients [10]. In particular, a 24-hour ambulatory BP measurement can exclude the bias of a measurer and is an effective method for assessing the variations within the day and BP load [11].

Thus, this study was designed to investigate whether a four-week intervention of essential oil inhalation using a simple, comfortable necklace is effective for reducing home BP, 24-hour ambulatory BP and salivary cortisol levels among patients with prehypertension or hypertension as a self-care intervention.

## 2. Methods

### 2.1. Study Design

We used a nonrandomized controlled design. The participants were allocated to one of three group, aromatherapy group (inhalation of essential oil), a placebo group (artificial fragrance), and a no-treatment control group.

### 2.2. Participants

Data collection for this experiment occurred over a seven-month period from February 2011 to August 2011. Prior to the beginning of the study, a review of the study design was performed by the bioethics committee board of the university, and their approval was obtained. Signed consent forms were obtained from all participants, who were informed that they may withdraw from the study at any point if they do not agree with the study. Possible side effects of the oil (e.g., nausea, vomiting, allergic reaction, and headache) during the treatment were explained. Participants showing an adverse reaction to the essential oil during treatment were excluded. After data collection, patients in the control group were provided with a necklace and essential oils to inhale. The inclusion criterion was having BP meeting JNC 7 standards for prehypertension and hypertension upon a physical exam in cooperation with family medicine. The participants were males and females with the ability to communicate, between 20 and 59 years of age, and with a known administration of antihypertensive medications; all participants agreed to the study. Exclusion criteria were history of prior nose surgery, a history of asthma or allergy to fragrance, a change in antihypertensive medication dose or type during the study period, and a presence of concurrent disease.

### 2.3. Sample Size

The sample size of this study was determined using G Power 3.1.2. An F-test was chosen as the method of analysis, and using a significance (*α*: 0.05), power (1 − *β*: 0.8), and effect size calculated from a prior study (0.34) [6], a total of 90 subjects were necessary.

### 2.4. Intervention

The experimental treatment of this study was the inhalation of essential oils. In consultation, with an international aroma therapist, four oils were thought to have an effect on BP and the autonomic nervous system—lavender (*Lavandula officinalis*), ylang-ylang (*Cananga odorata*), marjoram (*Origanum majorana*), and neroli (*Citrus aurantium*)—were blended at a 20 : 15 : 10 : 2 ratio and stored cold. Lavender alleviates cardiac excitation, lowers BP, and is effective in hypertension and palpitations. Ylang-ylang lowers BP, alleviates palpitations and nervous system excitation, and promotes emotional relaxation [12]. Marjoram lowers sympathetic nervous system activity and stimulates the parasympathetic nervous system, resulting in vasodilatation to reduce cardiac strain and decrease BP. Neroli brings forth emotional soothing and comfort and is effective in cardiac palpitations secondary to shock or fear [12]. 

As the method of intervention, the study group was provided with a necklace with the essential oils, while the placebo group was given a necklace with artificial fragrance. The subjects were instructed to wear the necklace during the daytime and place an aroma stone with two oil drops by the bedside for a 24-hour inhalation. 

To investigate the immediate effects of the essential oils, two drops of blended essential oils (study group) or artificial fragrance (placebo group) were dropped on an aroma stone between 10 AM and noon. The subjects sat in a comfortable position within 10 cm of the stone for two minutes of inhalation followed by three deep breaths. 

### 2.5. Outcome Measures

#### 2.5.1. Primary Main Outcomes

Home BP was measured twice each time prior to and 10 minutes after inhalation using a BP monitor with verified reliability (OMRON IA2, Japan). Subjects in the control group measured home BP twice, followed by 10 minutes without intervention, and repeated two BP measurements. Based on a study that serum levels of the main ingredients of lavender, linalool, and linalyl acetate are detected five minutes after massage with lavender and peak at 20 minutes [13], the stabilisation time after inhalation was considered, and home BP was measured twice after 10 minutes. BP was self-measured twice on the left arm between 10 AM and 12 PM in a location with room temperature between 18 and 24°C, in a sitting position after 5–10 minutes of rest. Ambulatory BP was measured using an ambulatory BP monitor (AND, Japan) over the span of 24 hours, with measurements every 30 minutes between 6 AM and 10 PM and every hour between 10 PM and 5:30 AM. After four weeks of intervention, all subjects from the study group, placebo group, and control group were tested for a 24-hour ambulatory BP and heart rate, and these measurements were compared against the values prior to the intervention; the salivary cortisol levels were also measured for all subjects.

#### 2.5.2. Secondary Main Outcomes

The salivary cortisol level was obtained by rinsing the mouth with cold water between 3 and 4 PM and spitting the saliva sample 10 minutes after the rinse (without swallowing the saliva) into a sputum sample collection bottle. The collected sample was immediately stored in a freezer (−20°C) and stored until analysis; the samples were sent in an ice box for transportation to N agency for analysis. The units of measurement were in *μ*g/dL, and the salivary cortisol level was analysed with an enzyme immunoassay (EIA) using equipment and media (ER HS SALIVARY CORTISOL, USA) designed for a microplate reader (USA). 

### 2.6. Data Analysis

Collected data were analysed using IBM SPSS 19.0 software. Homogeneity was verified using one-way ANOVA, and the treatment effect was analysed using one-way ANOVA, ANCOVA, Kruskal-Wallis' test, and repeated measures ANOVA.

## 3. Results

All 90 participants were assessed for eligibility, and none were excluded in the screening test. Over the study period of four weeks, two subjects dropped out from the study group and refused to undergo the 24-hour ambulatory BP measurement (refusal of data collection), three subjects were lost from the placebo group (two for the refusal of data collection and one for a change of residence), and two were lost from the control group (one for refusal of data collection and one for the change of residence), for a total of seven patients who dropped out of the study. Thus, the data were collected and analysed for 28 subjects in the study group, 27 subjects in the placebo group, and 28 subjects in the control group. 

The total number of participants in this study was 83 (28 in the study group, 27 in the placebo group, and 28 in the control group), with an average height of 168.9 cm and an average weight of 70.9 kg. The average age (in years) was 40 in the study group, 41.8 in the placebo group, and 40.1 in the control group, and the number of males in each respective group was 20 (71.4%), 19 (70.4%), and 20 (71.4%), showing no significant difference between the groups. In terms of marital status, there were 24 (85.7%), 19 (70.4%), and 24 (85.7%) married individuals in the study, placebo, and control groups, respectively, again without significant differences between groups. The participants used antihypertensive drugs were 3 of 28 in study group, 4 of 27 in placebo group, and 4 of 28 in the control group (Table 1). Homogeneity verification of the dependent variable showed no significant differences between the groups in home BP and 24-hour ambulatory BP. There was with a significant difference in salivary cortisol level between the groups (*F* = 3.14, *P* = .049).

### 3.1. Immediate Effects on BP

The SBP before and after inhalation over 8 measurements in 4 weeks decreased by 4.70 mm Hg from 132.3 mm Hg to 127.6 mm Hg after the inhalation of essential oils in the study group, increased by 0.97 mm Hg from 133.3 mm Hg to 134.2 mm Hg in the placebo group, and increased by 0.66 mm Hg from 133.2 mm Hg to 133.8 mm Hg in the control group. An analysis of pre and postintervention differences found a significant difference in SBP between the groups (*F* = 79.689, *P* < .001), and there was a meaningful reduction in the study group compared with the placebo and control groups from a posthoc analysis (*P* < .05) (Table 2).

The results of the eight DBP measurements over four weeks before and after inhalation in the study, placebo, and control groups identified a decrease of 1.21 mm Hg from 85.7 mm Hg to 84.5 mm Hg in the study group. There was an increase of 0.22 mm Hg from 83.8 mm Hg to 84.0 mm Hg in the placebo group and a reduction of 0.27 mm Hg from 85.0 mm Hg to 84.7 mm Hg in the control group. The changes in diastolic pressure before and after the inhalation in the three groups showed significant differences between them (*F* = 5.007, *P* = .009), and in a posthoc analysis, there was a significant reduction of BP in the study group compared with the placebo and control groups (*P* < .05) (Table 2).

The results of a subgroup analysis using prehypertensive and hypertensive subjects revealed significant differences in SBP of the prehypertensive (Kruskal-Wallis *χ* ^2^ = 22.479, *P* < .001) and hypertensive (*χ* ^2^ = 28.615, *P* < .001) patients between the study, placebo, and control groups (Table 3). 

The subgroup analysis revealed significant differences in DBP after essential oil inhalation between the study, placebo, and control groups only in the hypertensive subgroup (Kruskal-Wallis *χ* ^2^ = 8.223, *P* = .016) (Table 3).

### 3.2. Home BP

The SBP measurements obtained eight times over four weeks from all three groups using repeated measures ANOVA found no time-group interaction in the study group compared with other groups, but there was significance among the groups (*F* = 8.252, *P* = .001) (Table 4). Prior to the study treatment, the SBP results from an official physical examination were investigated. The values were 140.6 mm Hg for the study group, 141.0 mm Hg for the placebo group, and 140.1 mm Hg for the control group, showing no significant differences between the groups. The average BPs which were measured eight times over four weeks, two times before and after each treatment, are listed in Table 4. In the first week, pretreatment values were 136.6 mm Hg in the study group, 134.0 mm Hg in the placebo group, and 135.1 mm Hg in the control group, while posttreatment values were 131.8 mm Hg in the study group, 135.7 mm Hg in the placebo group, and 136.3 mm Hg in the control group, showing no significant difference among the three groups. By week 4, posttreatment values were 126.3 mm Hg in the study group, 132.9 mm Hg in the placebo group, and 134.0 mm Hg in the control group, with significant differences between the groups (*F* = 5.505, *P* = .006). The BP of the study group decreased to 10.3 mm Hg from the first week's pretreatment values. In the posthoc analysis, there was a significant decrease in BP in the study group compared with the placebo and control groups (Tukey, *P* < .05).

Repeated measures ANOVA results for the eight DBP measurements over four weeks of essential oil treatment revealed no significant difference in a time-group interaction or with time, or in groups (Table 4). An initial investigation before treatment using office DBPs revealed values of 87.4 mm Hg in the study group, 87.0 mm Hg in the placebo group, and 86.5 mm Hg in the control group, with no meaningful difference between the groups. The average of BPs measured eight times over four weeks, twice before and after each treatment, is shown in Table 4. In the first week, pretreatment values were 87.9 mm Hg in the study group, 84.8 mm Hg in the placebo group, and 87.0 mm Hg in the control group, and, by four weeks after treatment, the respective values were 83.8 mm Hg, 83.9 mm Hg, and 84.8 mm Hg. The changes were 4.1 mm Hg in the study group, 0.9 mm Hg in the placebo group, and 2.2 mm Hg in the control group, but these changes were not significantly different.

### 3.3. 24-Hour Ambulatory BP

The 24-hour ambulatory BP monitoring showed a decrease of 10.77 mm Hg after inhalation from 140.6 mm Hg to 129.9 mm Hg in the study group, an increase of 3.40 mm Hg from 132.6 mm Hg to 136.0 mm Hg in the placebo group, and a decrease of 0.71 mm Hg from 136.8 mm Hg to 136.1 mm Hg in the control group. The results of BP measurements before and after essential oil inhalation found a significant difference in SBP between the three groups (*F* = 16.150, *P* < .001), and a posthoc analysis revealed a significant difference in the study group in comparison with the placebo and control groups (Tukey, *P* < .05) (Table 5). The night time systolic pressure decreased by 2.61 mm Hg (from 121.1 mm Hg before inhalation to 118.5 mm Hg after inhalation) in the study group and by 0.88 mm Hg (from 116.4 mm Hg to 115.5 mm Hg) in the placebo group and increased by 3.86 mm Hg (from 114.8 mm Hg to 118.7 mm Hg) in the control group. There were no significant differences in night time SBPs pre and postinhalation between the three groups.

Daytime diastolic pressure decreased by 7.11 mm Hg from 90.5 mm Hg before essential oil inhalation to 83.3 mm Hg after inhalation in the study group, increased by 1.60 mm Hg from 84.0 mm Hg to 85.6 mm Hg in the placebo group, and decreased by 0.44 mm Hg from 87.8 mm Hg to 87.3 mm Hg in the control group. There was a significant difference in the daytime diastolic pressures pre and posttreatment between the three groups (*F* = 6.542, *P* = .002), and a posthoc analysis showed a significant decrease in the study group compared with the placebo and control groups (Tukey, *P* < .05). Night time DBP was increased after inhalation by 0.77 mm Hg, 0.23 mm Hg, and 1.96 mm Hg in the study, placebo, and control groups, respectively. There was no difference in night time diastolic pressure before and after essential oil inhalation between the groups.

### 3.4. Salivary Cortisol Concentration

The salivary cortisol concentration decreased by 0.02 *μ*g/dL from 0.16 *μ*g/dL before inhalation to 0.14 *μ*g/dL after inhalation in the study group, while it increased by 0.04 *μ*g/dL in the placebo group from 0.12 *μ*g/dL to 0.16 *μ*g/dL. The control group showed a change from 0.11 *μ*g/dL to 0.13 *μ*g/dL, for an increase of 0.02 *μ*g/dL. There was a significant difference in salivary cortisol changes before and after intervention between the three groups (*F* = 4.692, *P* = .012) (Table 6).

## 4. Discussion

This study aimed to identify the effect of a four weeks essential oil inhalation on the home BP, ambulatory BP, and salivary cortisol concentration in patients with hypertension and prehypertension.

As a result, the home BP decreased on average 4.70/1.21 mm Hg after inhalation in the study group, showing a significant decrease compared with the placebo and control groups. These results are consistent with the results of previous studies [4, 6, 14]. However, the magnitude of the difference was very low; therefore, the clinical significance can be deemed small despite the statistical significance. However, considering the results of a 5.5-year follow-up study by the Angio-Scandinavian Cardiac Outcomes Trial-BP-Lowering Arm (ASCOT-BPLA) that calcium channel blockers and ACE inhibitor combination therapy significantly lowers BP by 2.7/1.9 mm Hg and thus decreases the risk of cardiovascular death and stroke, even a small decrease in BP can have a clinical significance if long-term maintenance can be achieved [2].

A four-week home BP monitor shows repeated increases and decreases in BP before and after intervention and an overall decreasing trend. Thus, essential oil inhalation not only has an immediate BP-lowering effect, but also has a long-term effect. In this study, the screening office BP measured during the participant recruitment was higher than the home BP, which may be indicative of white coat hypertension. A four-week home BP monitoring to identify a long-term effect showed a decrease of 10.3/4.1 mm Hg in the study group, which is considered both statistically and clinically significant. 

In the subgroup analysis using prehypertension and hypertension groups, home SBP decreased in both the prehypertension (−4.96 mm Hg) and hypertension (−4.47 mm Hg) subgroups within the study group. DBP was significantly decreased only in the hypertension subgroup. A study by Hwang [6] showed decreased BP after aroma therapy consisting of lavender, ylang-ylang, and bergamot in stage 1 hypertension, while Jung [14] showed decreased BP and balanced autonomic nervous system activity with lavender aromatherapy in prehypertensive middle-aged women. This result is consistent with the results from a study by the Korean Society of Hypertension [2], which found that aerobic exercise decreased office BP by 3.0/2.4** **mm Hg and daytime ambulatory BP by 3.3/3.5** **mm Hg, with a larger BP-lowering effect in hypertensive subjects compared with normotensive population. A meta-analysis of 10 studies on aroma inhalation showed a small effect on systolic and SBPs [15], but the decrease of systolic and diastolic home BPs with essential oil inhalation achieved in this study have a large clinical significance.

A study by Jäger et al. [13] showed that after massage with lavender, the main ingredients of linalool and linalyl acetate were detected in the blood within five minutes and peaked at 20 minutes; by 90 minutes, they were mostly eliminated. Likewise, our experiment confirmed immediate BP lowering effects within 10 minutes of essential oil inhalation. As evidenced by Rimmer's study [16], which showed the presence of the ingredients of essential oil in blood within five minutes after application along with a reduction in stress reaction, the onset of the essential oil's effect occurs very rapidly and has an immediate effect on BP.

To identify the effect of essential oil inhalation after four weeks of treatment, a 24-hour ambulatory BP was measured and analysed during the day and the night. The daytime BP was decreased by 10.8/7.1 mm Hg and was significantly different from the placebo and control groups, while no difference was found on the night time BP. These results are consistent with previous research showing a decrease in BP of 6.7/3.0 mm Hg after three weeks of aromatherapy [4]. However, a study comparing eight weeks of triple and double pharmacotherapy using a 24-hour ambulatory BP showed a decrease of 30.3/19.7 mm Hg and 28.0/17.8 mm Hg in the daytime and night time BPs, respectively, in the triple therapy group and a decrease of 18.8–24.1 mm Hg/11.7–15.5 mm Hg in the daytime and 18.3–22.6/11.1–14.3 in the night time BPs in the double therapy group [17]. In comparison with these results, the effect of this complementary therapy is not large. However, according to a report that even a slight decrease in SBP (2 mm Hg) can lead to a decrease in mortality due to coronary artery disease and stroke by 7% and 10%, respectively [9], the average decrease in a 24-hour daytime systolic pressure of 10.77 mm Hg and diastolic pressure of 7.11 mm Hg can be considered clinically significant.

Despite the significant decrease in daytime BP found in this study, there was no significant decrease in night time BP, which contrasts with the significant decrease of both daytime and night time BPs in pharmacotherapy. We could not determine whether this difference is secondary to the use of the necklace during the daytime and aroma stones in night time or whether it is attributable to the differences in daytime and night time stress and autonomic nervous system hormones. Further research is required on this issue.

As seen in white-coat hypertension and office hypertension, to identify accurate BP and pulse of the patients, a 24-hour ambulatory BP monitoring is recommended rather than one or two BP measurements in the office. This study employed home BP and 24-hour ambulatory BP monitoring to identify the immediate and long-term effect of essential oil inhalation on decreasing BPs. 

Controlling BP requires a strong will of the patient, but for a more universal approach, that is, an easy to use, self-administered, and economical approach, essential oil inhalation can be used as an effective intervention for BP control. 

In this study, the concentration of salivary cortisol after the inhalation of essential oils in prehypertensive and hypertensive patients was decreased significantly in comparison with the placebo group (exposed to artificial fragrance) and the control group (no interventions). A study by Hwang [6] showed a significant change in the serum cortisol level in the study group after four weeks of aroma inhalation, while Jung [14] reported decreased activity of the sympathetic nervous system and increased activity of the parasympathetic nervous system after five days of aromatherapy, aiding in the balance of the autonomic nervous system. In particular, Kim [18] reported that the inhalation group showed the most significant decrease in serum cortisol compared with the massage group. Although Seo [19] found that aromatherapy using a necklace during the daytime for one week did not result in decreased concentration of salivary cortisol, the levels were decreased significantly when the study group was exposed to a second week of inhalation after a two-week washout period. But the cortisol difference between the pretest and the posttest was so small, further research was needed to clarify the study effects.

Of the essential oils used in this study, marjoram decreases the activity of the sympathetic nervous system and stimulates the parasympathetic system [12]. Because the cortisol levels were increased in the placebo and control groups (in comparison with the decreased levels in the study group) in this study, the continuous essential oil therapy may have activated the parasympathetic system by controlling stressors. However, because the values were obtained before the study and four weeks after the experiment, the exact difference in cortisol concentration could not be identified, and what effect the four-week study duration had on changes in cortisol concentration could not be confirmed. 

Despite an increased risk of stroke and death from cardiovascular disease in the absence of other risk factors when measured BPs are in the prehypertension or hypertension range, hypertension is often poorly managed due to its mild subjective symptoms. As such, the inhalation of essential oil, which brings out relaxation effects and stress and immunologic response control in hypertensive patients, has an immediate and chronic BP effect as observed through home monitoring and 24-hour ambulatory BP monitoring rather than a single measurement. 

This study was conducted without considering the subjects' preference for certain types of essential oils, and although the subjects had varied responses to the aroma, there was no adverse effect or repulsion towards it. Over the four-week period, the participants were actively involved in the treatment, and by self-monitoring home BP, they were able to acknowledge their BP and observe the therapeutic effects, leading to the increased willingness to control their BP.

To summarise the discussion thus far, there are many complementary therapies being researched for the management of stress and BP. Although research in aromatherapy has been approached from many angles to provide scientific evidence, there is a little current clinical use of aromatherapy in patients with hypertension or stress. To facilitate its use, a short-term administration such as a one-time inhalation or an easier, comprehensive, and patient-centred approach is required. 

## 5. Conclusion

This work is a quasi-experimental study using a nonequivalent control group, a nonsynchronised design to identify the effects of essential oil inhalation on home BP, a 24-hour ambulatory BP, and salivary cortisol levels. Both immediate and long-term effects were identified from this study. Although a 24-hour ambulatory daytime BP was decreased significantly, no differences were found in night time BPs. In addition, decrease in salivary cortisol concentration was noted. A relaxation therapy using essential oils for BP control to prevent the progression of hypertension is strongly recommended, in particular, in the simple and convenient form of a necklace. The essential oil therapy is also believed to function as a promising nursing intervention.

## Figures and Tables

**Table 1:** Homogeneity test before treatment among participants.

Characteristics	Aromatherapy (*n* = 28)	Placebo (*n* = 27)	Control (*n* = 28)	*F* or *χ* ^2^	*P*
Height (cm)	170.4 ± 8.7	166.6 ± 8.6	169.5 ± 8.8	1.48	.23
Weight (kg)	70.6 ± 13.1	70.4 ± 10.9	71.7 ± 11.6	0.09	.91
Age (yr)	40.0 ± 7.1	41.8 ± 8.6	40.1 ± 7.4	0.46	.64
Medications					
Yes	3	4	4	0.24	.89
No	25	23	24		
Office BP (mmHg)					
Systole	140.6 ± 6.2	141.0 ± 6.2	140.1 ± 6.9	0.12	.88
Diastole	87.4 ± 7.5	87.0 ± 8.3	86.5 ± 7.9	0.09	.91
Home BP (mmHg)					
Systole	136.6 ± 8.8	134.0 ± 9.3	135.1 ± 10.5	0.52	.60
Diastole	87.9 ± 6.5	84.8 ± 9.3	87.0 ± 9.0	1.04	.36
Perceived stress	79.0 ±15.5	75.5 ± 8.2	73.6 ± 10.9	1.07	.35
Salivary cortisol (*μ*g/dL)	0.16 ± 0.09	0.12 ± 0.06	0.11 ± 0.06	3.14	.05

Values are expressed as mean ± standard deviation; BP: blood pressure.

**Table 2:** The effect of essential oil on home BP at 10 minutes after inhalation.

BP	Group	Pretest	Posttest	Difference (posttest and pretest)	*F*	*P*
	Aromatherapy (*n* = 28)	132.3 ± 5.66	127.6 ± 5.70	−4.70 ± 1.93^a^		
Systole	Placebo (*n* = 27)	133.3 ± 8.48	134.2 ± 8.86	0.97 ± 1.68^b^	79.689	<.001
	Control (*n* = 28)	133.2 ± 6.92	133.8 ± 7.03	0.66 ± 2.01^b^		

	Aromatherapy (*n* = 28)	85.7 ± 5.44	84.5 ± 5.42	−1.21 ± 1.51^a^		
Diastole	Placebo (*n* = 27)	83.8 ± 7.35	84.0 ± 8.03	0.22 ± 1.94^b^	5.007	.009
	Control (*n* = 28)	85.0 ± 6.94	84.7 ± 7.13	−0.27 ± 1.64^b^		

Values are expressed as mean ± standard deviation; Different superscripts (a, or b) indicate a significant difference (Tukey's test; *P* < .05); BP: blood pressure.

**Table 3:** Subgroup analysis of home SBP after inhalation.

	Group	Pretest	Posttest	Difference (posttest and pretest)	Kruskal-Wallis *χ* ^2^	*P*
SBP						
	Aromatherapy (*n* = 13)	130.8 ± 5.65	125.8 ± 6.00	−4.96 ± 1.89^a^		
Prehypertension	Placebo (*n* = 10)	130.0 ± 7.91	130.3 ± 8.10	0.33 ± 1.62^b^	22.479	<.001
	Control (*n* = 14)	130.8 ± 7.45	131.4 ± 7.37	0.55 ± 2.35^b^		
						
	Aromatherapy (*n* = 15)	133.5 ± 5.54	129.1 ± 5.14	−4.47 ± 2.00^a^		
Hypertension	Placebo (*n* = 17)	135.2 ± 8.42	136.5 ± 8.69	1.35 ± 1.64^b^	28.615	<.001
	Control (*n* = 14)	135.5 ± 5.66	136.3 ± 5.94	0.78 ± 1.68^b^		

DBP						
	Aromatherapy (*n* = 13)	84.1 ± 5.00	83.2 ± 5.30	−0.95 ± 1.67		
Prehypertension	Placebo (*n* = 10)	82.9 ± 6.47	83.0 ± 6.98	0.08 ± 1.79	2.511	.285
	Control (*n* = 14)	82.9 ± 6.88	82.7 ± 6.83	−0.23 ± 1.73		
						
	Aromatherapy (*n* = 15)	87.2 ± 5.58	85.7 ± 5.43	−1.45 ± 1.38^a^		
Hypertension	Placebo (*n* = 17)	84.4 ± 7.97	84.7 ± 8.73	0.30 ± 2.08^b^	8.223	.016
	Control (*n* = 14)	87.0 ± 6.63	86.7 ± 7.12	−0.31 ± 1.62^b^		

Values are expressed as mean ± standard deviation; Different superscripts (a, or b) indicate a significant difference (Tukey's test; *P* < .05); BP: blood pressure.

**Table 4:** The effects of essential oil inhalation on Home BP for 4 weeks.

		SBP (mm Hg)				DBP (mm Hg)			
		Aromatherapy (*n* = 28)	Placebo (*n* = 27)	Control. (*n* = 28)	*F* (*P*)	Aromatherapy (*n* = 28)	Placebo (*n* = 27)	Control. (*n* = 28)	*F* (*P*)
Screening office BP		140.6 ± 6.2	141.0 ± 6.2	140.1 ± 6.9	0.12 (.88)	87.4 ± 7.5	87.0 ± 8.3	86.5 ± 7.9	0.09 (.91)

Home BP									

1st week									
(Tuesday)	Before	136.6 ± 8.8	134.0 ± 9.3	135.1 ± 10.5	0.52 (.60)	87.9 ± 6.5	84.8 ± 9.3	87.0 ± 9.0	1.04 (.36)
	After	131.8 ± 8.3	135.7 ± 9.3	136.3 ± 9.1	2.14 (.125)	86.6 ± 6.4	84.5 ± 10.5	86.0 ± 8.1	0.43 (.65)
(Friday)	Before	134.3 ± 8.6	135.8 ± 12.0	133.4 ± 7.8	0.44 (.65)	87.3 ± 8.5	84.5 ± 9.1	85.3 ± 8.7	0.78 (.46)
	After	128.3 ± 7.2^a^	136.3 ± 10.3^b^	134.9 ± 8.8^b^	6.42 (.003)	85.5 ± 6.7	84.9 ± 9.0	86.1 ± 9.1	0.14 (.87)
2nd week									
(Tuesday)	Before	133.6 ± 10.3	132.3 ± 11.4	131.4 ± 9.9	0.31 (.74)	86.6 ± 7.3	83.9 ± 8.1	84.2 ± 8.3	1.03 (.36)
	After	128.6 ± 8.4	133.4 ± 11.8	132.6 ± 9.3	1.86 (.16)	86.6 ± 5.6	84.3 ± 8.9	83.8 ± 7.8	1.16 (.32)
(Friday)	Before	133.4 ± 5.4	132.0 ± 9.6	134.5 ± 9.0	0.64 (.53)	85.7 ± 6.6	83.8 ± 7.7	85.3 ± 8.9	0.47 (.63)
	After	127.8 ± 6.4^a^	133.7 ± 9.6^b^	134.9 ± 9.4^b^	5.50 (.006)	84.2 ± 7.2	83.9 ± 7.7	85.2 ± 9.3	0.19 (.83)
3rd Week									
(Tuesday)	Before	129.1 ± 6.8	132.8 ± 12.4	132.2 ± 9.2	1.16 (.32)	85.3 ± 6.6	83.1 ± 9.6	83.7 ± 9.1	0.51 (.60)
	After	125.6 ± 7.3^a^	134.2 ± 11.3^b^	132.1 ± 9.4^b^	6.15 (.003)	83.5 ± 7.2	82.4 ± 10.5	84.6 ± 9.4	0.42 (.66)
(Friday)	Before	129.3 ± 8.2	132.6 ± 9.6	133.6 ± 9.1	1.77 (.18)	84.4 ± 7.7	82.9 ± 8.7	85.2 ± 7.9	0.57 (.57)
	After	125.7 ± 6.0^a^	132.3 ± 9.7^b^	133.7 ± 10.1^b^	6.54 (.002)	83.5 ± 6.3	83.6 ± 8.7	83.4 ± 8.6	0.002 (1.00)
4th week									
(Tuesday)	Before	131.4 ± 6.6	134.2 ± 10.4	132.2 ± 8.9	0.73 (.49)	83.9 ± 6.8	83.8 ± 9.8	83.7 ± 8.3	0.003 (1.00)
	After	126.4 ± 6.5^a^	135.4 ± 12.1^b^	132.3 ± 7.9^b^	7.00 (.002)	82.5 ± 7.8	84.9 ± 11.1	83.6 ± 7.5	0.45 (.61)
(Friday)	Before	130.4 ± 8.1	132.5 ± 12.4	133.2 ± 9.5	0.56 (.57)	84.8 ± 6.9	83.9 ± 11.7	85.2 ± 8.0	0.14 (.87)
	After	126.3 ± 6.3^a^	132.9 ± 12.9^b^	134.0 ± 8.0^b^	5.51 (.006)	83.8 ± 7.8	83.9 ± 10.8	84.8 ± 9.2	0.11 (.90)

*F* (*P*)		Time: 0.70 (.67); Group: 8.25 (.001); G*T: 1.24 (.25)				Time: 0.84 (.56); Group: 0.09 (.91); G*T: 0.92 (.54)			

Mean ± SD: mean ± standard deviation. *Repeated measures of ANOVA. Different superscripts (a, or b) indicate a significant difference (Tukey's test; *P* < .05).

**Table 5:** The effects of essential oil inhalation on ambulatory BP among the groups.

BP	Group	Pre-test	Post-test	Difference (posttest and pretest)	*F*	*P*
SBP						
	Aromatherapy (*n* = 28)	140.6 ± 10.56	129.9 ± 10.81	−10.77 ± 8.95^a^		
Day time*	Placebo (*n* = 27)	132.6 ± 7.91	136.0 ± 9.48	3.40 ± 8.48^b^	16.2	<.001
	Control (*n* = 28)	136.8 ± 12.31	136.1 ± 10.79	−0.71 ± 10.96^b^		
						
	Aromatherapy (*n* = 28)	121.1 ± 15.33	118.5 ± 13.73	−2.61 ± 10.57		
Night time**	Placebo (*n* = 27)	116.4 ± 13.46	115.5 ± 15.76	−0.88 ± 14.44	1.9	.160
	Control (*n* = 28)	114.8 ± 11.61	118.7 ± 11.41	3.86 ± 13.60		

DBP						
	Aromatherapy (*n* = 28)	90.5 ± 9.12	83.3 ± 8.01	−7.11 ± 7.13^a^		
Day time*	Placebo (*n* = 27)	84.0 ± 5.80	85.6 ± 8.81	1.60 ± 8.95^b^	6.5	.002
	Control (*n* = 28)	87.8 ± 9.41	87.3 ± 8.91	−0.44 ± 11.50^b^		
						
	Aromatherapy (*n* = 28)	74.6 ± 8.82	75.4 ± 8.47	0.77 ± 7.60		
Night time**	Placebo (*n* = 27)	72.1 ± 8.52	72.3 ± 11.20	0.23 ± 10.13	0.2	.783
	Control (*n* = 28)	71.5 ± 8.50	73.4 ± 8.24	1.96 ± 10.18		

Values are expressed as mean ± standard deviation.

Different superscripts (a, or b) indicate a significant difference (Tukey's test; *P* < .05).

*Daytime BP: blood pressure measured from 6 AM to 9:30 PM.

**Night time BP: blood pressure measured from 10 PM to 5:30 AM.

**Table 6:** The effects of essential oil inhalation on salivary cortisol (*μ*g/dL) among the groups.

Group	Pretest	Posttest	Difference (posttest and pretest)	*F*	*P*
Aromatherapy (*n* = 28)	0.16 ± 0.09	0.14 ± 0.05	−0.02 ± 0.06^a^		
Placebo (*n* = 27)	0.12 ± 0.06	0.16 ± 0.09	0.04 ± 0.09^b^	4.69	.012
Control (*n* = 28)	0.11 ± 0.06	0.13 ± 0.07	0.02 ± 0.08^b^		

Values are expressed as mean ± standard deviation.

Different superscripts (a, or b) indicate a significant difference (Tukey's test; *P* < .05).
